# CDK5RAP3 Participates in Autophagy Regulation and Is Downregulated in Renal Cancer

**DOI:** 10.1155/2019/6171782

**Published:** 2019-04-02

**Authors:** Jun Li, Xinyi Hu, Ming Su, Hongliang Shen, Wei Qiu, Ye Tian

**Affiliations:** ^1^Department of Urology, Beijing Friendship Hospital, Capital Medical University, Beijing, China; ^2^Department of Clinical Laboratory, Peking University People's Hospital, Beijing, China

## Abstract

Renal cancer is one of the most common malignant urological tumors; however, its diagnosis and treatment are not well established. In the present study, we identified that CDK5 regulatory subunit-associated protein 3 (CDK5RAP3), a putative tumor suppressor in many cancers, was downregulated in renal cancer tissues. Through loss- and gain-of-function experiments, we observed that the action of CDK5RAP3 in renal cancer cells was different in Caki-1 and 769-P cell lines. Knockdown of endogenous CDK5RAP3 in Caki-1 slightly increased cell viability, whereas overexpression of CDK5RAP3 in 769-P cells inhibited cell viability. In addition, we observed that CDK5RAP3 participated in the regulation of autophagy in renal cancer. Knockdown of CDK5RAP3 induced significant inhibition of autophagy in Caki-1 cells but not in 769-P cells. In contrast, overexpression of CDK5RAP3 significantly activated autophagy in 769-P cells, as evidenced by increased LC3-II levels. However, the LC3-II could not be altered by CDK5RAP3 overexpression in Caki-1 cells. These findings demonstrated that CDK5RAP3 is downregulated in renal cancer and may be associated with autophagy.

## 1. Introduction

Renal cancer is one of the most common urological cancers worldwide. Since it does not present early symptoms and is typically diagnosed at an advanced stage, many patients are unable to receive radical or partial nephrectomies. According to the histological categorization of renal cancer, renal cell carcinoma (RCC) comprises nearly 80-90% of all renal cancers [1]. The most common pathological category of RCC is clear cell renal cell carcinoma. Surgical resection is the primary therapy for early diagnosis patients, but for advanced patients, the 5-year life expectancy is significantly reduced to roughly 10% [2]. Although targeted therapy drugs, such as sorafenib and sunitinib, have already been used as a first-line therapy for advanced renal cancer, resistance to these drugs is inevitable and always leads to anticancer therapy failure. Therefore, finding novel molecular targets that are associated with tumor growth and progression is critical to the exploration of new therapies for this disease.

CDK5 regulatory subunit-associated protein 3 (CDK5RAP3), an important intracellular factor related to proliferation and apoptosis, has been reported to be associated with many cancers such as colorectal cancer, hepatocellular cancer, breast cancer, lung cancer, gastric cancer, and head and neck squamous cell carcinoma [3–8]. CDK5RAP3 has been reported to bind with RelA and suppress the NF-*κ*B pathway through the LXXLL/leucine zipper-containing alternative reading frame (ARF) to repress head and neck squamous cell carcinoma [8, 9]. CDK5RAP3 regulates apoptosis through the G2/M DNA damage check point [10] or induces rupture of the nuclear envelope [11]. Recently, low expression of CDK5RAP3 and its partner DDRGK1 has been correlated with poor prognosis of gastric cancer [12]. These effects may be associated with the inhibition of Wnt/*β*-catenin signaling by inhibiting AKT phosphorylation [13]. However, the function of CDK5RAP3 in cancers remains controversial. In hepatocellular cancer, CDK5RAP3 was reported to promote metastasis through PAK4 activation [4]. These data indicate that the function of CDK5RAP3 varies in different cancers. However, there have been no reports on the relationship between CDK5RAP3 and renal cancer until recently.

Autophagy is a key process governing the degradation of long-lived proteins and organelles and mediates many important biological processes, such as self-renewal, metabolism, energy generation, and cell death [14–16]. It has been reported that many diseases are associated with autophagy [17–19]. However, the role of autophagy in cancer remains unclear and mostly relies on the origin of the primary cancer. Adaptive activation of autophagy protects the cancer cells against adverse conditions. In contrast, maladaptive autophagy induces cell injury and cell death. It has been reported that cancer cells are able to activate autophagy when exposed to chemotherapy drugs [20–22]. In renal cancer, sorafenib could induce autophagic cell death through Akt inhibition [23]. However, blocking autophagy with chloroquine enhances the anticancer effect of sunitinib [24]. These evidences indicate that autophagy is precisely regulated in the cell; however, the mechanism is not fully clarified. In the present study, we observed that CDK5RAP3 was downregulated and regulated cell viability in renal cancer. More importantly, we found that CDK5RAP3 participated in the regulation of autophagy in renal cancer.

## 2. Materials and Methods

### 2.1. Renal Cancer Patients

The studies that related to patients were approved by the Ethics Committee of Beijing Friendship Hospital, Capital Medical University, according to the Declaration of Helsinki. Twenty-five patients diagnosed with clear cell renal cell carcinoma in Beijing Friendship Hospital were enrolled in this study. All of the patients underwent partial or radical nephrectomy. The cancerous tissues were cut and immediately submerged in liquid nitrogen or fixed in 4% paraformaldehyde solution for further analysis. Clinical staging for each patient was evaluated following the TNM-staging system. Histologic diagnosis was performed by a pathologist, and the tumor grading was scored according to the Fuhrman system.

### 2.2. Immunohistochemistry Staining

Tissues fixed in 4% paraformaldehyde solution were embedded in paraffin and cut into 4 *μ*m sections. The sections were dewaxed, serially rehydrated and incubated in 3% H_2_O_2_ to block endogenous peroxidases. Then, the sections were incubated with a diluted rabbit anti-CDK5RAP3 primary antibody (Abcam) at 4°C overnight. The sections were then washed 3 times with PBS and incubated with HRP/Fab polymer-conjugated secondary antibody (ZSGB-BIO) for 30 min at room temperature. After the incubation, the sections were washed and visualized using a diaminobenzidine system.

### 2.3. Cell Culture and Treatment

Human renal cancer cell lines, Caki-1, Caki-2, 786-O and 769-P, were obtained from the National Infrastructure of Cell Line Resource (Beijing, China). Caki-1 and Caki-2 cells were cultured in Mycos 5A medium; 786-O and 769-P cells were in RPMI-1640 medium, both containing 10% fetal bovine serum (FBS) in an atmosphere of 5% CO_2_ at 37°C. For loss-of-function studies, the cells were transfected with two different Silencer™ Select siRNAs (s37159 and s37161, cat. 4392420; Thermo Fisher) or a negative control (Thermo Fisher) with Lipotransfectamine 3000 (Thermo Fisher) for 48 h. For overexpression, an adenoviral vector carrying CDK5RAP3 was used, and adenovirus carrying GFP was employed as a control. Cells were harvested for further analysis 48 h postinfection.

### 2.4. Cell Viability Analysis

Cell viability was determined using a Cell Counting Kit-8 (CCK-8, Dojindo, Kumamoto, Japan) assay. Briefly, a density of 1 × 10^4^ cells/well was seeded onto a 96-well plate. A final concentration of 10% (*v*/*v*) of CCK-8 reagent was added into the medium and incubated at 37°C for 2 h. The relative optical density was read at 450 nm.

### 2.5. Protein Extraction and Analysis

Total protein from tissue or cellular samples was harvested, quantified, and denatured. Equal masses (50 *μ*g) of protein from each sample were loaded for SDS polyacrylamide gel electrophoretic separation and blotted onto nitrocellulose membranes. The membranes were then blocked with 5% nonfat milk at room temperature for 1 h and incubated at 4°C overnight with specific primary antibodies: rabbit anti-CDK5RAP3, rabbit anti-LC3B (Abcam, Cambridge, MA, USA), and mouse anti-GAPDH (Santa Cruz, Dallas, TX, USA). Then, the membranes were washed with TBST and incubated with HRP-conjugated goat anti-rabbit or mouse secondary antibodies (ZSGB-BIO, Beijing, China) for 1 h at room temperature. The membranes were then washed with TBST, and the specific bands were visualized using SuperSignal West Femto Maximum Sensitivity Substrate (Pierce, Rockford, IL, USA). The intensity of each band was calculated using Quantity One software V 4.6.2 (Bio-Rad, Hercules, CA, USA), and GAPDH was employed as an internal control.

### 2.6. Statistical Analysis

All statistical procedures were performed using SPSS 19.0 software (SPSS Inc., Chicago, IL, USA). A two-tailed Student's *t*-test or Mann-Whitney *U* test was used for analyzing differences between two groups. *P* < 0.05 was considered as statistically significant.

## 3. Results

### 3.1. CDK5RAP3 Is Decreased in Renal Cancer

**Figure 1:**
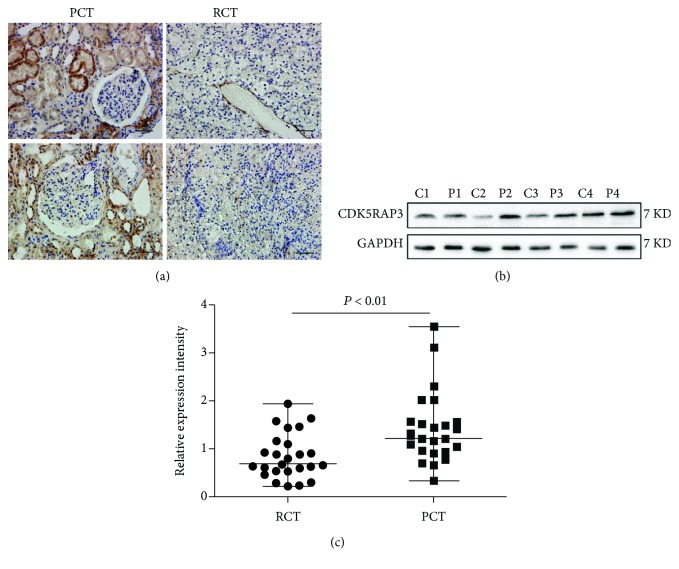
CDK5RAP3 is downregulated in renal cancer. (a) Immunohistochemical staining of CDK5RAP3 was performed in renal cancerous (RCT) and paracancerous (PCT) tissues. Representative images are shown. (b, c) The protein level of CDK5RAP3 was detected by western blotting (b). GAPDH was employed as an internal control, and representative images are shown (*n* = 25; C: cancerous tissue, P: paracancerous tissue). The relative expression intensity of CDK5RAP3 was measured, and a Mann-Whitney *U* test was used for analyzing differences between RCT and PCT.

To explore the role of CDK5RAP3 in renal cancer, we recruited 25 renal cancer patients diagnosed as clear cell renal cell carcinoma from histologic results. The detailed profiles for all patients tested are listed in the Supplementary material file 1. We first compared CDK5RAP3 expression in renal cancerous and paracancerous tissues. By using immunohistochemical staining, we observed that CDK5RAP3 was strongly expressed in renal tubule epithelial cells of paracancerous tissues. In contrast, the staining intensity of CDK5RAP3 in renal cancer was weaker compared with the paracancerous tissues (Figure 1(a)). By using western blotting, we compared the relative intensity of CDK5RAP3 in cancerous and paracancerous tissues. We found that the expression intensity of CDK5RAP3 in renal cancer tissues was reduced to 56.1% of the paracancerous tissues (Figures 1(b) and 1(c)).

### 3.2. CDK5RAP3 Is a Potential Tumor Suppressor in Renal Cancer

**Figure 2:**
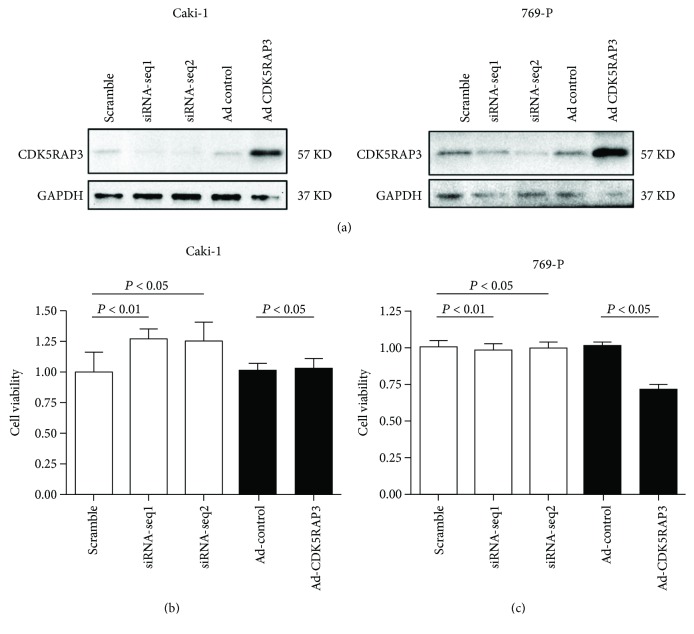
CDK5RAP3 inhibits renal cancer cell viability. (a) The expression of CDK5RAP3 in Caki-1 and 769-P cells is shown. (b, c) Caki-1 (a) and 769-P (b) cells were transfected with siRNAs targeting CDK5RAP3 with two different sequences (seq1 and seq2) for knockdown or were infected with adenoviruses carrying CDK5RAP3 for overexpression. CCK-8 assays were performed to measure cell viability. Data are expressed as mean ± standard deviation, *n* = 6 per group.

To explore the function of CDK5RAP3 in renal cancer, we initially detected the expressions of CDK5RAP3 in four commonly used renal cancer cell lines: 769-P, 786-O, Caki-1, and Caki-2 cells (Supplementary material file 2). We employed 769-P and Caki-1 cells for further study. Then, we performed CCK-8 assays in Caki-1 and 769-P cells, respectively, by overexpression or knockdown of endogenous CDK5RAP3. The efficiency of CDK5RAP3 knockdown and overexpression are shown in Figure 2(a). We observed that knockdown of the endogenous CDK5RAP3 expression slightly enhanced the cell viability of Caki-1 cells; however, exogenous overexpression of CDK5RAP3 did not vary the cell viability (Figure 2(b)). In contrast, knockdown of CDK5RAP3 in 769-P cells did not vary cell viability, whereas overexpression caused a significant decrease in viability (Figure 2(c)). These data indicated that CDK5RAP3 showed different behaviors in Caki-1 and 769-P cells.

### 3.3. CDK5RAP3 Regulates Autophagy in Renal Cancer Cells

**Figure 3:**
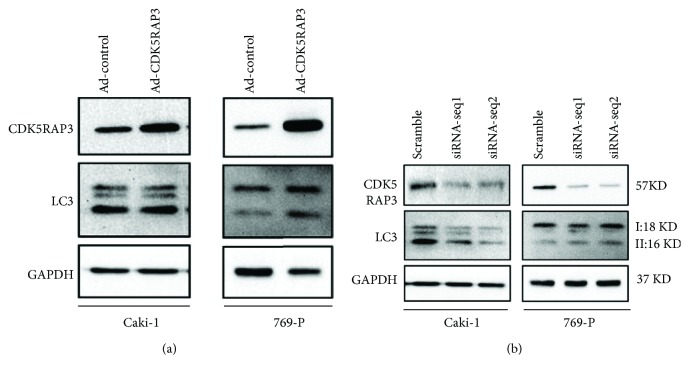
CDK5RAP3 regulates autophagy in renal cancer cells. (a, b) Endogenous CDK5RAP3 in Caki-1 and 769-P cells was infected with adenoviral vectors carrying CDK5RAP3 for overexpression (a) or knocked down (b) with two specific siRNAs (seq1 and seq2). The conversion of LC3 was detected using western blotting.

Autophagy plays an important role in the regulation of proliferation, cell death, and metabolism in cancer [25, 26]. However, currently there is no study that reports the association between CDK5RAP3 and autophagy. We induced overexpression or knockdown of endogenous CDK5RAP3 expression in Caki-1 and 769-P cells for 48 h, and the conversion of LC3 was detected. We found that knockdown of CDK5RAP3 in Caki-1 cells led to a decrease in LC3 conversion (Figure 3(a)). In 769-P cells, knockdown of CDK5RAP3 did not vary LC3 conversion, but overexpression induced a significant upregulation of LC3-II (Figure 3(b)). These results indicated that CDK5RAP3 regulated autophagy differently in Caki-1 and 769-P cells.

## 4. Discussion

Due to their insensitivity towards radiotherapy and chemotherapy, the prognosis for advanced renal cancer patients is often quite poor. Thanks to the use of targeted therapy drugs, such as sorafenib and sunitinib, the survival rate of the disease has been enormously improved; however, drug resistance is frequently inevitable. Therefore, identification of differentially expressed genes in cancer tissues is helpful in the exploration of novel therapeutic targets for the disease. In the present study, we found that the expression of CDK5RAP3 was downregulated in renal cancer tissues and may participate in the regulation of cell viability and autophagy in renal cancer. These findings indicated that CDK5RAP3 might be a potential target for renal cancer therapy.

The encoding gene of *CDK5RAP3* is located on the long arm of chromosome 20. It has been reported that mutations in this gene were correlated with the late onset of colorectal cancer [3]. Loss of CDK5RAP3 expression was found in a portion of human head and neck squamous cancers, whereas genetic knockdown of CDK5RAP3 enhances the invasion activity through inhibition of the NF-*κ*B pathway [8]. In gastric cancer, CDK5RAP3 acts as a tumor suppressor through inactivation of Wnt/*β*-catenin signaling [6, 13], and downregulation of this protein in gastric cancer indicates poor prognosis [12]. CDK5RAP5 can be cleaved by caspases and induces the abnormal microtubule bundling and rupture of the nuclear envelop and thus promotes apoptosis [11]. However, data from hepatocellular cancer indicates CDK5RAP3 promotes metastasis through the activation of PAK4 [4, 9]. These results indicate that CDK5RAP3 may play different roles according to the origin of the tumor. In the present study, we observed that CDK5RAP3 was highly expressed in paracancerous tissues, especially in the renal tubule epithelial cells. However, CDK5RAP3 was significantly downregulated in renal cancer compared with paracancerous renal tissues. Knockdown of CDK5RAP3 in Caki-1 cells slightly increased cell viability. However, we observed comparable effects on cell viability in Caki-1 cells with CDK5RAP3 overexpression and in 769-P cells with CDK5RAP3 knockdown. These results support that CDK5RAP3 is a potential tumor suppressor in renal cancer.

The role of autophagy is known to play important roles in cancer cells. Autophagy can be activated during stress conditions. Adaptive activation of autophagy protects the cancer cells against adverse conditions and contributes to the resistance towards chemotherapy and molecular targeted drugs [14–16]. However, severe activation of autophagy, known as maladaptive autophagy, could directly induce cell death [17–20]. Therefore, the level of autophagy is strictly controlled by various signaling pathways, such as PI3K/Akt/mTOR and ERK1/2 [23]. Blockade of autophagy in many cancers (including renal cancer cells) induces the exhaustion of metabolic substrates and causes energetic impairment, which eventually leads to cell death [27]. Therefore, chloroquine, as an inhibitor of autophagy, is a potential novel antitumor drug [28]. In renal cancers, activation of autophagy with mTOR inhibitor everolimus shows antitumor effects, whereas combination of chloroquine to block the fusion of autophagosomes and lysosomes potentiates the antitumor effects of everolimus [29]. These results indicated that both activation and inhibition of autophagy could exhibit antitumor effects. Our data indicate that knockdown of endogenous CDK5RAP3 in Caki-1 cells caused a significant reduction of the autophagy level. Overexpression of CDK5RAP3 in 769-P cells resulted in the activation of autophagy, which indicated that CDK5RAP3 might participate in the regulation of autophagy in renal cancer. However, we currently do not understand why Caki-1 and 769-P cells responded differently on autophagy upon exogenously altering CDK5RAP3 expression. We postulated that this might rely on the different regulation of autophagy in these cells, because the LC3-II/LC3-I ratios in Caki-1 cells appeared higher than in 769-P cells. However, the detailed mechanism and the consequence of CDK5RAP3 on autophagy regulation remain to be investigated.

Taken together, our data demonstrated that the expression of CDK5RAP3 was downregulated in renal cancer compared with the paracancerous tissues. CDK5RAP3 is a potential tumor suppressor and participates in autophagy regulation in renal cancer, which suggests CDK5RAP3 could be a candidate biomarker for individualized treatment.

## 5. Conclusions

Renal cancer is one of the most common urological tumors worldwide; however, its prognosis has not been sufficiently addressed. In the present study, we found that CDK5RAP3 was downregulated in renal cancer and participated in the regulation of apoptosis and cell viability. More importantly, for the first time, we reported that CDK5RAP3 is associated with autophagy. Our findings indicate that CDK5RAP3 may be a novel biomarker of renal cancer.

## Data Availability

The original data used to support the findings of this study are included within the supplementary information file.

## Supplementary Materials

Supplementary Materials The clinical characteristics of all the subjects.
