# Supplementary figures and images for: CDK5RAP3 Participates in Autophagy Regulation and Is Downregulated in Renal Cancer
Source: Dis Markers. 2019 Apr 2;2019:6171782. doi: 10.1155/2019/6171782 (PMC6466961; doi:10.1155/2019/6171782)

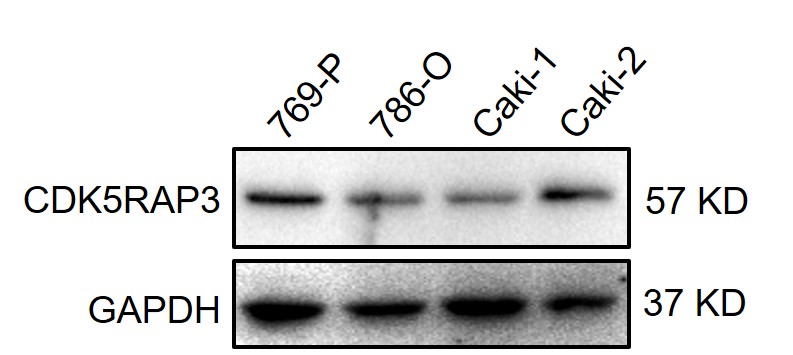


Supplementary figure. CDK5RAP3 expressions in four commonly used renal cancer cell lines.

Supplement: Supplementary Materials — The clinical characteristics of all the subjects. [file 6171782.f1.zip › Supplementary material file 2.docx]
